# Wolfram-like syndrome with bicuspid aortic valve due to a homozygous missense variant in *CDK13*

**DOI:** 10.1038/s10038-021-00922-0

**Published:** 2021-04-21

**Authors:** Anushree Acharya, Syed Irfan Raza, Muhammad Zeeshan Anwar, Thashi Bharadwaj, Khurram Liaqat, Muhammad Akram Shahzad Khokhar, Jenna L. Everard, Abdul Nasir, University of Washington Center for Mendelian Genomics, Deborah A. Nickerson, Michael J. Bamshad, Muhammad Ansar, Isabelle Schrauwen, Wasim Ahmad, Suzanne M. Leal

**Affiliations:** 1 Center for Statistical Genetics, Gertrude H. Sergievsky Center, and the Department of Neurology, Columbia University Medical Center, New York, NY USA; 2 Department of Biochemistry, Faculty of Biological Sciences, Quaid-i-Azam University, Islamabad, Pakistan; 3 Department of Biochemistry, HBS Medical and Dental College, Islamabad, Pakistan; 4 Department of Biochemistry, CMH Kharian Medical College, Punjab, Pakistan; 5 Major Shabbir Sharif Shaheed Hospital, THQ Level, Kunjah, Gujrat, Punjab Pakistan; 6 Synthetic Protein Engineering Lab (SPEL), Department of Molecular Science and Technology, Ajou University, Suwon, South Korea; 7 Department of Genome Sciences, University of Washington, Seattle, WA USA; 8 Department of Pediatrics, University of Washington, Seattle, WA USA; 9 Taub Institute for Alzheimer’s Disease and The Aging Brain, Columbia University Medical Center, New York, NY USA

**Keywords:** Consanguinity, Genetic linkage study

## Abstract

**Background:**

Wolfram syndrome (WFS) is characterized by deafness, diabetes mellitus, and diabetes insipidus along with optic atrophy. WFS has an autosomal recessive mode of inheritance and is due to variants in *WFS1* and *CISD2*.

**Methods:**

We evaluated the underlying molecular etiology of three affected members of a consanguineous family with hearing impairment, bicuspid aortic valve, diabetes mellitus and insipidus, clinodactyly, and gastrointestinal tract abnormalities via exome sequencing approach. We correlated clinical and imaging data with the genetic findings and their associated phenotypes.

**Results:**

We identified a homozygous missense variant p.(Asn1097Lys) in *CDK13*, a gene previously associated with autosomal dominant congenital heart defects, dysmorphic facial features, clinodactyly, gastrointestinal tract abnormalities, intellectual developmental disorder, and seizures with variable phenotypic features.

**Conclusion:**

We report a homozygous variant in *CDK13* and suggest that this gene causes an autosomal recessive disorder with hearing impairment, bicuspid aortic valve, diabetes mellitus and insipidus, clinodactyly, and gastrointestinal tract abnormalities.

## Introduction

Wolfram syndrome (WFS) is a neurodegenerative disease that is characterized by diabetes insipidus and diabetes mellitus with optical atrophy, and deafness (DIDMOAD) and has a prevalence of 1 in ~500,000 children [1]. Both genes responsible, i.e., *WFS1*, for Wolfram syndrome type 1, and *CISD2*, for Wolfram syndrome type 2, are inherited with an autosomal recessive (AR) mode of inheritance and encode for wolframin and CDGSH iron-sulfur domain 2 proteins, respectively, and have high expression in brain, lung, heart, and pancreas [2, 3].

In addition to diabetes mellitus and insipidus with optical atrophy, and hearing impairment (HI), individuals with variants in *WFS1* also can present with intellectual development disorder, and cerebellar ataxia, along with osseous, renal, and cardiac abnormalities [1, 4–6]. In almost all patients with WFS1, onset of sensorineural (SN) HI is between 5 and 39 years of age, progressive optic atrophy between 6 weeks and 19 years, and diabetes insipidus and diabetes mellitus with average ages of onset between 6 and 14 years, respectively [7]. WFS2, has all the clinical features of WFS1 with the exception of diabetes insipidus and it is also characterized by bleeding peptic ulcers and irregular platelet aggregation with onset as early as 6 years of age [1, 8]. *WFS1* also causes autosomal dominant (AD) nonsyndromic progressive low-frequency SNHI [9, 10] and AD Wolfram-like syndrome (WFSL; OMIM: 614296) that is characterized by congenital progressive low- and middle-frequency SNHI, diabetes mellitus, and optic atrophy [11]. Some patients with WFSL also present with severe neuropsychiatric symptoms including depression, and anxiety [12, 13]. Additional disorders that have similar etiology as WFS are AR Thiamine-responsive megaloblastic anemia syndrome, AR Alstrom syndrome, AR classic Refsum Disease, AR Mohr–Tranebjaerg syndrome, and X-linked Charcot-Marie-Tooth disease type 5.

We report on a consanguineous Pakistani family with an AR WFS-like phenotype that segregates a *CDK13* missense variant. The three affected family members, who are children, have severe to profound SNHI affecting all frequencies, diabetes mellitus and insipidus, bicuspid aorta, clinodactyly, and gastrointestinal (GI) tract abnormalities. There were no signs of intellectual disability or optic atrophy. Genotyping was performed on all available DNA samples, i.e., three affected and three unaffected family members. Neither homozygosity nor linkage was observed within the regions containing the *WFS1* and *CISD2* genes. Exome sequencing of a DNA sample from an affected family member revealed a homozygous missense variant, c.3291 C > A: p.(Asn1097Lys) in *CDK13* [NM_003718.5], which lies within the region of homozygosity. Sanger sequencing of DNA samples from the remaining family members demonstrated that the variant segregated with the syndrome and had a LOD score of 3.11.

*CDK13*, expressed ubiquitously in human tissues, is part of the cyclin-dependent kinase family that regulates gene transcription, alternative splicing of RNA, and C-terminal domain (CTD) phosphorylation, along with CDK12 and cyclin K [14, 15]. Heterozygous variants in *CDK13* are known to cause congenital heart defects, dysmorphic facial features, and intellectual developmental disorder (CHDFIDD) as well as GI tract abnormalities, clinodactyly, and seizures. The majority of reported variants in *CDK13* are de novo [16–18]. This is the first report of an AR phenotype of a family presenting with WFS-like phenotype segregating *CDK13*.

## Materials and methods

### Patient recruitment

**Fig. 1:**
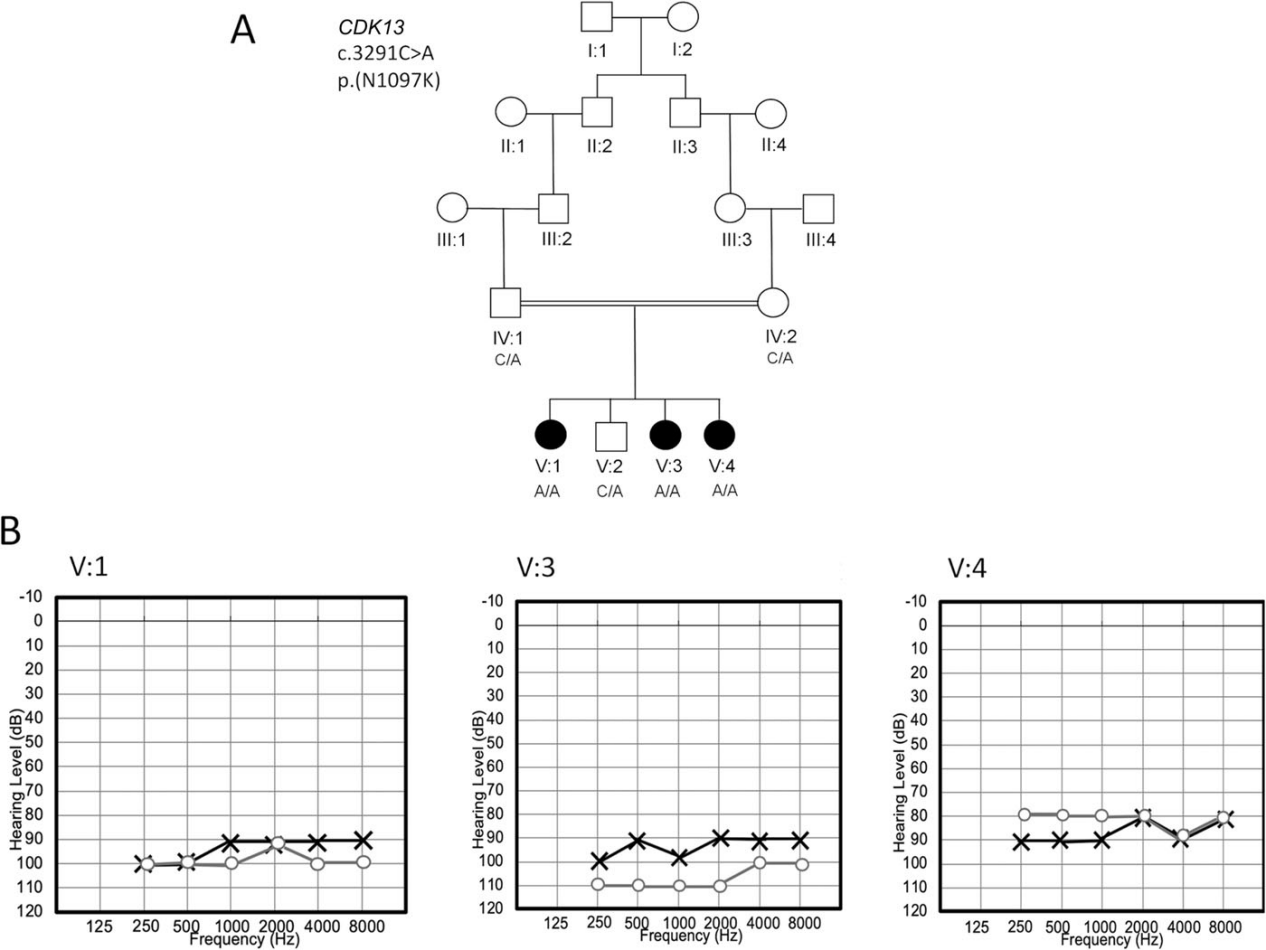
Pedigree and audiograms for affected family members. **a** Pedigree drawing of family 4743. Squares represent males and circles females. Solid symbols signify that the individual presents with Wolfram-like syndrome and clear symbols are unaffected family members. A DNA sample from affected family member V:3 underwent exome sequencing. For each family member with an available DNA sample the genotypes are displayed for the p.(Asn1097Lys) variant. **b** Pure-tone audiograms of hearing-impaired family members V:1, V:3, and V:4, each illustrating bilateral HI

Institutional review board (IRB) approval for human research was obtained from IRB committees at Quaid-i-Azam University (IRB-QAU-153) and Columbia University (IRB-AAAS2343). Written informed consent was obtained from adult family members of pedigree 4743 and the parents consented for their four children after they provided their assent. Blood samples were obtained from three affected and three unaffected family members (Fig. 1a) and DNA was extracted using a phenol-chloroform procedure [19]. Of the six members in this consanguineous family, three of four children (V:1, V:3, and V:4) presented with SNHI, diabetes mellitus and insipidus, bicuspid aortic valve, clinodactyly, and GI tract abnormalities. The twin brother of V:1 was stillborn. The parents (father IV:1 and mother IV:2) and one male sibling (V:2) do not present with any symptoms and their medical histories are unremarkable.

### Clinical Assessment

A full physical examination was performed for two (V:3 and V:4) of the three affected children who presented with SNHI. A panel of blood tests to evaluate diabetes status, liver, renal, and serum chemistry were also performed for V:3 and V:4. The third affected child, V:1, died at 12¾ years of age, and phenotype information is limited to medical records and reports from her parents. Pure-tone audiometry was performed on affected family members V:1, V:3, and V:4. Tympanometry was performed on V:3 and V:4. Ophthalmoscopy and visual acuity tests were also performed on V:3 and V:4.

Small bowel biopsy and esophagogastroduodenoscopy were performed on V:4 due to her history of chronic diarrhea and failure to thrive. Orthopedic and x-ray assessments of V:3 and V:4 hands were performed. Echocardiography (ECHO) was performed on all affected children (V:1, V:3, and V:4) to evaluate structural heart defects, and electrocardiography (ECG) was performed on V:3 and V:4 to evaluate for any functional defects.

### Homozygosity mapping and linkage analyses

Genotyping was performed using the Infinium HumanCore-24 v1.0 BeadChip (Illumina, Inc., San Diego, California, USA), which has 307,342 markers, on all individuals with available DNA samples. Using this data and PLINK [20], the sex of each individual and pedigree relationships were verified.

Using HomozygosityMapper [21] and genotype data for all family members, homozygous regions in the genome were identified. Superlink-Online SNP 1.1 [22] was used to perform multipoint and two-point linkage analysis using a fully penetrant AR mode of inheritance and allele frequencies obtained from gnomAD for the South Asian population. Multipoint linkage analysis was performed using the genotyped markers spanning the regions of *WFS1* and *CISD2* genes. Two-point linkage analysis was performed and LOD scores were calculated using genotype data obtained for the identified variant.

### Sanger and exome sequencing

Exome sequencing was performed on a DNA sample obtained from affected child V:3 using libraries prepared with the NimblegenV2 library preparation kit (Roche, Basel, Switzerland), following the manufacturer’s protocol. Sequencing was performed by 100 bp paired-end sequencing on a HiSeq2500 instrument (Illumina Inc, San Diego, California, USA) with a mean on-target coverage of 39.5×. Filtered reads were aligned to the Human genome (Hg19/GRC37) using the Burrows–Wheeler transform method (BWA-MEM) [23]. Reads were sorted, and PCR duplicates were marked using Picard. Base quality recalibration and insertion/deletion (InDel) realignments were performed using the Genome Analysis Toolkit (GATK) [24]. Single nucleotide variants (SNVs) and InDel variants were called jointly with HaplotypeCaller and recalibrated with GATK [25, 26].

Annotation and filtering were performed using ANNOVAR [27] and custom scripts, including prediction scores from dbNSFP and dbscSNV, to evaluate missense and splice site variants, respectively [26, 28]. Homozygous and potentially compound heterozygous variants with a population-specific minor allele frequency (MAF) < 0.005 in the Genome Aggregation Database (gnomAD) [29] and Kaviar Database of Genomic Variants (Kaviar) [30] were selected and prioritized based on their annotation. Copy number variants (CNVs) were also assessed using CONiFER (v0.2.2) [31] and CNVs with a MAF < 0.005 in the Database of Genomic Variants (DGV) were retained [32].

To validate and test for segregation of candidate variants discovered through exome sequencing, Sanger sequencing was performed using Polymerase Chain Reaction (PCR) followed by direct sequencing of the PCR product using both forward and reverse primers, on an ABI3130XL sequencer (Applied Biosystems Inc., Foster City, California, USA).

### In silico RNA expression analysis in the inner ear

Several publicly available datasets were used for an in silico investigation of the expression of *Cdk13* during various developmental stages of mouse inner ear development.

Using previously generated datasets present in the Shared Harvard Inner-Ear Laboratory Database (SHIELD) [33], we also studied *Cdk13* expression during mouse inner ear development. The first dataset detailed expression over developmental stages E16, P0, P4, and P7 [34]. Data were obtained from the cochlea and utricles of mice that expressed enhanced green fluorescent protein (EGFP) under the Pou4f3 promoter [34]. Fluorescence-activated cell sorting (FACS) was used to separate hair and surrounding cells prior to RNA extraction [34]. These data were supplemented with a second dataset containing expression data over developmental stages E12, E13, E16, P0, P6, and P15 for the spiral and vestibular ganglion [35].

Finally, *Cdk13* expression in single cells of the cochlear epithelium during mouse developmental stages E14, E16, P1, and P7 obtained from the gene Expression Analysis Resource (gEAR) [36] were visualized [37]. Data were generated through single-cell RNA sequencing of CD-1 mouse embryos [37]. *Cdk13* expression was also grouped based on cell types into four overarching classes i.e., developing supporting cells, developing prosensory cells, developing sensory cells, and developing greater epithelial ridge cells [37].

### Homology modeling

In silico modeling was performed to evaluate any structural changes in the protein due to variant p.(Asn1097Lys). Crystal structure of Human IKK2 (PDB ID: 4E3C) [38] with the highest sequence and structural similarity with the target sequence was selected for modeling [39]. PDB ID:4E3C shares ∼23% amino acid sequence identity with Human *CDK13*.

## Results

### Clinical presentation

The parents observed all three affected children having HI in early childhood. Pure-tone audiometry testing performed at 250–8000 Hz is available for all affected siblings, which was performed for V:1 at 9 years of age, V:3 at 10 years of age, and V:4 at 15 years of age (Fig. 1b). Across all tested frequencies, affected children V:1 and V:3 had profound bilateral SNHI and V:4 had severe to profound bilateral SNHI. Individual V:3 was recently administered hearing aids at 12 years of age, V:4 has been using hearing aids since she was 12 years of age, and V:1 at the time of her death was not using hearing aids. Tympanometry for siblings V:3 and V:4 showed normal compliance and pressure with overall normal development of the middle ear.

Individuals V:1 and V:3 were diagnosed with diabetes mellitus at 2 years of age, while V:4 was diagnosed at 2.5 years of age. Before her death, V:1 was administered insulin regularly. At the time of last examination both V:3, age 17 years, and V:4, age 13 years, were receiving regular doses of 70/30 insulin and had HbA1c levels of 10.6 and 10.7, respectively. Affected member V:4 has hyper-urination. In addition, V:1, V:3, and V:4 suffered from nocturnal enuresis until the ages of 11, 10, and 6, respectively, indicative of diabetes insipidus.

Blood test results for V:3 and V:4 showed elevated platelet count, lymphocytes, eosinophils, and monocytes while their neutrophils were decreased (Supplementary Table 1). In addition, V:4 had elevated white blood cell count and V:3 had anisopoikilocytosis along with few reactive lymphocytes. D-dimer levels for platelet aggregation in V:3 and V:4 were within normal limits. At recent examination, fasting blood glucose levels for V:3 and V:4 were 137 mg/dl and 234 mg/dL, respectively. Serum C-peptide levels for both V:3 and V:4 are below 0.03 ng/mL. Biochemical assessments of renal electrolytes were within normal range for V:4, but her liver alkaline phosphatase was elevated more than two-fold while her globulins were slightly elevated. The liver albumin:globulin ratio for V:4 was slightly decreased. For V:1, liver alanine aminotransferase (ALT) was raised to ~600 U/L, and aspartate transaminase (AST) was raised to 295 U/L at the time of death. On her death certificate, the cause of death is listed as liver failure. It was also suspected that she had celiac disease. Affected family member V:4 is also on a gluten-free diet since her tissue transglutaminase antibodies (tTG-IgA) of 28 U/mL is indicative of celiac disease. Liver function tests for V:3 and V:4 were otherwise normal (Supplementary Table 1).

All affected children, V:1, V:3, and V:4 present with a bicuspid aortic valve, which was detected by ECHO. For V:3 and V:4, echocardiograms showed that normal biventricular systolic function was retained, and no aortic regurgitation, or stenosis was observed (Supplementary Fig. 1). In addition, all cardiac septa were normal and no atrioventricular ventriculoarterial discordance was observed. Heart rate of all affected siblings were within normal range (90–95 beats/minute) and tachycardia was not suspected. At the time of her death at 12¾ years of age, V:1 also suffered a cardiac arrest and cardiopulmonary resuscitation was performed under medical care. Electrocardiograms performed on the two surviving siblings V:3 and V:4 showed no additional heart defects (Supplementary Fig. 2).

**Fig. 2:**
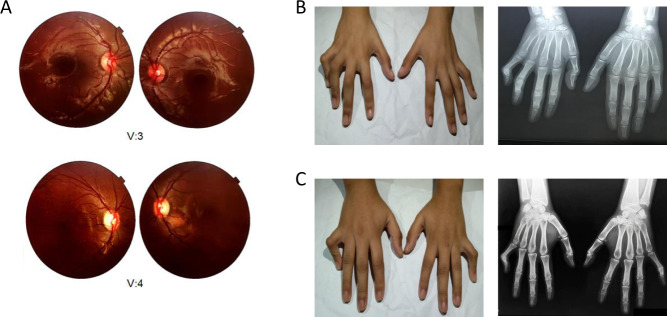
Fundoscopy and hand x-rays and photographs for two affected family members. **a** Fundoscopy images for V:3 (left) and V:4 (right). **b** Hand photograph and x-ray image for V:3 suggest clinodactyly due to shortening of middle phalanges of bilateral fifth and fourth digits resulting in radial curvature of little fingers and the fourth digit of the right hand toward the right. **c** Hand photograph and x-ray image for V:4 suggest clinodactyly due to shortening of middle phalanges of bilateral fifth digits resulting in radial curvature to little fingers toward the right

It was recommended that V:3 wear corrective lenses due to hyperopia at 12 years of age, which had progressed slightly since her last examination (right eye: +2.25 D; left eye: +1.25 D). Eye examination of individual V:4 showed normal vision. According to the parents, V:1 did not have any vision problems or wear corrective lenses. Nystagmus was not observed in any of the affected siblings. Fundoscopy results for V:3 and V:4 were within normal limits and no optic atrophy was observed (Fig. 2a). For affected family member V:4, 3D optical coherence tomography images were also normal (Supplementary Fig. 3).

In addition to liver failure that is listed as cause of death on her death certificate, V:1 also presented with cardiac arrest and hematemesis due to a severe GI tract ulcer. Affected family members V:1, V:3, and V:4 all had gastritis, steatorrhea, and diarrhea. Individual V:4 also had a history of failure to thrive, stomach cramps, and severe constipation. Histopathological examination of duodenal cap in V:4 revealed mild blunting villi and increased levels of intraepithelial lymphocytes with no evidence of inflammation, metaplasia, atrophy, or lymphoid aggregates.

Affected family member V:1 had a history of severe seizures due to epilepsy. However, so far, no seizures have been observed for individuals V:3 or V:4.

Radiology reports for V:3 displayed clinodactyly with shortening of middle phalanges of bilateral fifth and fourth digits resulting in radial curvature of little fingers and the fourth digit of the right hand toward the right (Fig. 2b). The x-ray images for V:4 also showed clinodactyly due to shortening of middle phalanges of bilateral fifth digits resulting in radial curvature to little fingers toward the right (Fig. 2c). For both V:3 and V:4, no acute fractures, dislocations, bone, regional soft tissue or phalangeal joint abnormalities have been diagnosed. Following orthopedic evaluation of V:3 and V:4, finger splints were suggested. Parents report joint angulation of limb digits for V:1, but images are unavailable.

Unaffected family members IV:1, IV:2, V:2 did not present any of the symptoms and abnormalities observed in the affected members. Lastly, no developmental delay, regression of speech, autism, or facial dysmorphism were observed in any affected sibling. V:1 was in Grade 5 preceding her death, V:3 and V:4 attend school and are working toward their pre-O and O levels, respectively. The phenotypes observed for individuals V:1, V:3, and V:4, and their overlap with traits for WFS-1, WFS-2, WFSL, and CHDFIDD can be found in Table 1.

**Table 1:** An overview of all phenotypes observed in affected members of the family 4743 and overlap with WFS-1, -2, WFSL, and CHDFIDD

	V:1	V:3	V:4	WFS-1	WFS-2	WFSL	CHDFIDD
Mode of inheritance	AR	AR	AR	AR	AR	AD	AD
SNHI	Yes	Yes	Yes	Yes	Yes	Yes	No
Diabetes mellitus	Yes	Yes	Yes	Yes	Yes	Yes	No
Diabetes insipidus	Yes	Yes	Yes	Yes	No	No	No
Platelet aggregation	No	No	No	No	Yes	No	No
Cardiac septal defect	Yes	Yes	Yes	No	No	No	Yes
Optic atrophy	No^a^	No^a^	No^a^	Yes	Yes	Yes	No
GI tract abnormalities	Yes	Yes	Yes	No	Yes	No	Yes
Clinodactyly	Yes	Yes	Yes^b^	No	No	No	Yes
Seizures/Epilepsy	Yes	No	No	Yes	No	No	Yes
ID/DD	No	No	No	Yes	No	No	Yes

*WFS-1* Wolfram syndrome-1, *WFS-2* Wolfram syndrome-2, *WFSL* Wolfram syndrome-like, *CHDFIDD* Congenital heart defects, dysmorphic facial features, and intellectual developmental disorder, *AR* Autosomal recessive, *AD* Autosomal dominant, *GI* Gastrointestinal, *ID/DD* Intellectual disability/developmental disorder.

^a^Within the age of onset for optic atrophy.

^b^Reported by the parents of V:1, but no radiology was performed.

### Exome sequencing, homozygosity mapping, and linkage analyses

Homozygosity mapping of genotype data with 307,342 markers revealed six regions of homozygosity in the three affected children compared to the three unaffected family members in (Supplementary Table 2). Known Wolfram syndrome genes *WFS1* and *CISD2* did not lie within the regions of homozygosity. Negative parametric multipoint LOD scores were obtained spanning both the *WFS1* and *CISD2* genes (data not shown). *CDK13* which lies on chromosome 7p14.1 is within the region of homozygosity. Using exome sequence data from individual V:3, candidate variants obtained from filtering as well as Wolfram syndrome genes *WFS1* and *CISD2* were evaluated via the Integrative Genomics Viewer (IGV) [40]. Any coding regions for *WFS1* and *CISD2* that were not covered by at least 10 reads were Sanger sequenced in all affected family members to ensure no variants in these genes went undetected. Next, candidate variants in other genes were validated (Supplementary Table 3) and tested for segregation in all family members with an available DNA sample. Only one gene with a homozygous missense variant [NM_003718.5, c.3291 C>A: p.(Asn1097Lys)] in *CDK13* within the region of homozygosity (i.e, chr7:106,157,161–130,629,493) segregated with the phenotype. No compound heterozygous variants or CNVs that could potentially contribute to the disease phenotype were identified.

**Fig. 3:**
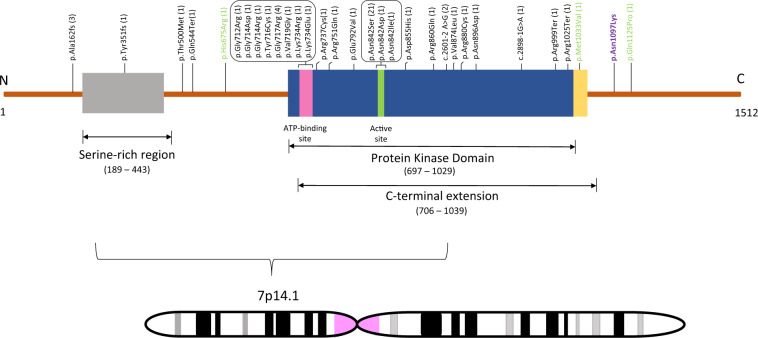
All variants reported in *CDK13* transcript NM_003718.4. The number shown in parentheses indicates the number of times the variant has been previously reported. Variants shown in black are pathogenic or likely pathogenic, those in green are of unknown significance, and the one in purple segregates in family 4743

The p.(Asn1097Lys) variant in *CDK13* is present in the homozygous region, chr7p14.1 and has a LOD score of 3.11. The variant is rare and has an overall MAF of 6.365 × 10^−5^ and a MAF of 4.9 × 10^–4^ in South Asians in gnomAD, with no observation in the homozygous state in any database. The variant is predicted as deleterious by several bioinformatic tools and is conserved between species [CADD = 29; GERP = 4.63]. It is located just C-terminally of the protein kinase domain (Fig. 3). Heterozygous variants in the protein kinase domain *CDK13* are known to cause congenital heart defects, along with dysmorphic facial features, and intellectual development disorder. There are no known phenotypes with AR inheritance due to variants in *CDK13*. However, because of the phenotypic overlap with AD WFS, the predicted damaging effect, and segregation of this variant with the phenotype in this family, this variant is the likely cause of the AR WFS-like phenotype.

### Single-cell RNA expression of *Cdk13*

**Fig. 4:**
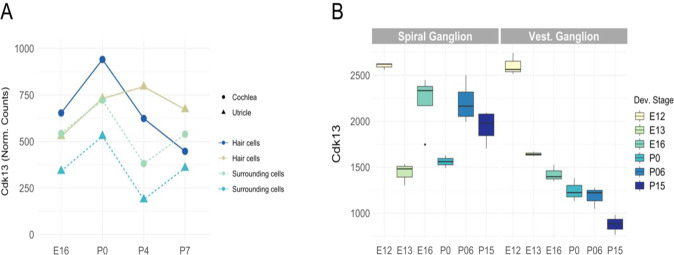
Expression of *Cdk13* in mouse cochlea and vestibular system during development. **a** Normalized counts of *Cdk13* RNA expression data in hair cells and surrounding cells from the cochleae and utricles of mice at four developmental stages: E16, P0, P4, and P7. *Cdk13* is expressed in cochlea and utricle cells for the four developmental stages. **b** *Cdk13* expression data from RNA microarrays in spiral ganglion neurons and vestibular ganglion neurons from mice collected at six developmental stages: E12, E13, E16, P0, P06, and P15. Expression data is based on perfect match and mismatch probe differences (PM/MM). *Cdk13* has the highest expression in E12 in both ganglions. Data were obtained from SHIELD (Shared Harvard Inner-Ear Laboratory Database), further processed, and plotted with R

In mice, *Cdk13* is expressed in cochlea and utricle cells over four developmental stages: E16, P0, P4, and P7. In cells surrounding the cochlea and utricle, highest levels of expression were seen at P0. In hair cells, highest expression levels were seen at stage P0 in cochlea and at P4 in utricle (Fig. 4a). An upregulation of *Cdk13* is observed for inner hair cells from E16 through P7. While an upregulation of *Cdk13* is also seen in outer hair cells, it is only observed until P1 after which it is downregulated through P7. Examining microarray expression data for *Cdk13* in spiral and vestibular ganglion over stages E12, E13, E16, P0, P06, and P15 of mice, we found that *Cdk13* alternates being upregulated and downregulated in spiral ganglion, but in vestibular ganglion *Cdk13* is continuously downregulated from E12 to P15 (Fig. 4b).

Overall, single-cell expression data obtained from gEAR shows widespread expression of *Cdk13* in cochlear floor epithelia over four developmental stages of CD-1 mice, i.e., E14, E16, P1, and P7 (Supplementary Fig. 4). This data also shows a proportional increase in both intensity of *Cdk13* expression and the number of cells expressing *Cdk13* in the OHC and IHC across the four stages, suggesting that it may play a role in inner ear sensory epithelia development (Supplementary Figs. 5, 6).

### Homology modeling

**Fig. 5:**
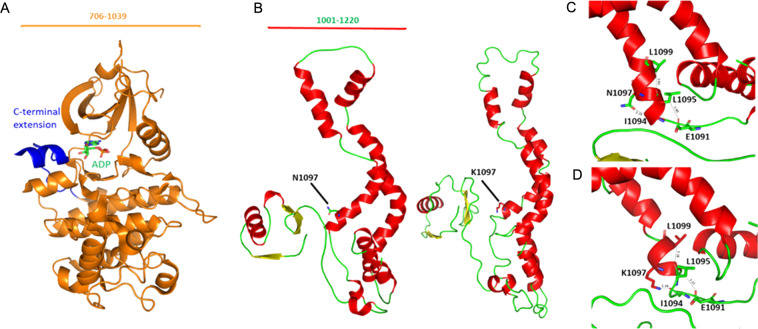
Homology modeling of *CDK13*. **a** The crystal structure of kinase domain (PDB ID: 5EFQ). The kinase domain is shown in orange and the C-terminal domain in blue. The number of amino acids visualized is indicated at the top. **b** Predicted protein models of the wild‐type and mutant with asparagine replaced with lysine. The number of amino acids visualized is indicated at the top. **c**. Asparagine residue at α-helix is predicted to be interacting with residue Ile1094 whereas in the mutant protein. **d** In the mutant protein, Asparagine is replaced by lysine p.(Asn1097Lys) which modifies the native bond interactions distance. Due to the difference in interaction with nearby residues resulted in shortening of the α-helix

The crystal structure of *CDK13* (PDB ID: 5EFQ) is displayed in Fig. 5. In the wild-type protein, an asparagine residue at 1097 interacts with the isoleucine residue at 1094 of the α-helix. Whereas, in the mutant protein, the substitution of a lysine residue at 1097 modifies the native bond interaction distance. This resulted in shortening of the α-helix due to difference in interaction with other residues nearby.

## Discussion

We performed whole-exome sequencing and identified a homozygous missense variant in *CDK13* that segregates with a Wolfram-like syndrome in a consanguineous Pakistani family. The three affected family members present with severe to profound bilateral SNHI, insulin-dependent diabetes mellitus and insipidus, bicuspid aortic valve, clinodactyly, and GI tract abnormalities. One affected member also presented with seizures due to epilepsy. Although Wolfram syndrome was suspected, all affected individuals also had bicuspid aortic valves with no other structural and functional heart defects as well as clinodactyly which are inconsistent with WFS-1 and −2. All three affected siblings are within the age of onset of optic atrophy. Therefore, it cannot be ruled out that the two living affected family members will develop optic atrophy as they grow older, although typically WFS patients have onset <15 years of age, while for AD Wolfram-like syndrome onset of optic atrophy has been reported to occur as late as 20 years of age [13, 41].

Overlapping features observed between family 4743 and CHDFIDD include cardiac septal defects, clinodactyly, and GI tract abnormalities that were observed in the three affected siblings [42]. Seizures due to epilepsy, observed only in affected member V:1, have also been described for some patients with CHDFIDD.

*CDK13* is ubiquitously expressed in human tissues with highest levels of expression in the cerebellum, thyroid gland, colon, skin, ovary, and testis [43, 44]. It is also expressed in the heart, duodenum, liver, spleen, lymph nodes, and bone marrow among other tissues [43]. Fetal brain, liver, and muscle tissues derived from glioblastoma cDNA libraries also showed high levels of *CDK13* expression [45]. In mice, *Cdk13* is strongly expressed in the retina, testes, ovary, uterus, gallbladder, heart, thyroid gland, and kidney [16]. In silico single-cell RNA expression data from mice shows that *Cdk13* is prominently expressed in mice inner ear from developmental stage E16 to P7, with varying levels of expression observed in the spiral and vestibular ganglion from stages E12 to P15 as well.

De novo pathogenic mutations in *CDK13*, implicated in CHDFIDD with varying phenotypes, are clustered in the protein kinase domain that binds ATP and magnesium [17, 18, 42, 46]. Thus far, 56 cases with *CDK13* heterozygous pathogenic, likely pathogenic or variants of unknown significance (VUS), mostly loss of function (LoF) variants (missense, splice site, nonsense, and frameshift) with wide phenotypic heterogeneity have been evaluated [47]. Notably, only three of these cases are of unknown inheritance due to lack of availability of one or both parents while the rest are de novo [48]. The majority of CHDFIDD variants (86%) lie in the highly conserved protein kinase domain and four have been observed more than once, i.e., p.(Gly717Arg) (*N* = 4), p.(Asn842Ser) (*N* = 21), p.(Ala162fs) (*N* = 3), and c.2601–2 A>G (*N* = 2) [17, 49], with only p.(Ala162fs) lying outside of the protein kinase domain. Missense variant [c.3291 C>A, p.(Asn1097Lys)] segregating in family 4743 lies outside the conserved protein kinase domain. To the best of our knowledge, it is also the first *CDK13* variant to be associated with an AR phenotype. Most AD *CDK13* variants have been predicted to lead to a LoF, although functional studies have not been performed to validate the classification. So far, of the 42 known cases presenting AD CHDFIDD due to *CDK13* variants one patient has only mild or no ID that was confirmed by Wechsler Nonverbal (WNV-IQ) test [42]. Given the location of the p.(Asn1097Lys) variant outside of the protein kinase domain and the AR presentation of the phenotype, we hypothesize that it has a hypomorphic effect, causing a partial loss in gene function, but additional functional analyses will need to be performed to confirm the variant’s effect on the gene and protein.

Embryonic lethality was observed in homozygous Cdk13^tm1a/tm1a^ knockout mice after stage E15.5 due to chronic heart failure, suggesting their role in transcriptional regulation during embryonic development [16]. Heart defects, such as pericardial effusion and ventricular wall thinning due to decreased myocardium, were also detected in Cdk13^tm1a/tm1a^ knockout mice embryos along with underdeveloped brain, lung, liver, and kidney [16]. Gross morphology of the kidneys and liver of Cdk13^tm1a/tm1a^ knockout mice between E14.5 and E16.5 was severely affected leading to kidney failure, while liver function remained intact [16]. Notably, in the same study, heterozygous Cdk13^tm1a/+^ mice were normal and healthy. In a study of Cdk13^tm1b(EUCOMM)Hmgu^ mice, preweaning lethality with complete penetrance was observed for homozygous Cdk13^tm1b(EUCOMM)Hmgu^ mice, while heterozygous Cdk13^tm1b(EUCOMM)Hmgu^ mice displayed heightened startle reflex, abnormal lens morphology, and cataracts [50].

In conclusion, this study has identified a novel homozygous missense variant, c.3291 A>C: p.(Asn1097Lys), in *CDK13* segregating in a Pakistani family with severe to profound SNHI, bicuspid aortic valve, diabetes mellitus and insipidus, clinodactyly, and GI tract abnormalities which suggests that besides CHDFIDD, *CDK13* also likely causes an AR Wolfram-like syndrome which includes cardiac defects, clinodactyly, and GI tract abnormalities. The identification of additional families with AR Wolfram-like syndrome due to *CDK13* will aid in determining the complete disease spectrum and also serve to confirm the role of *CDK13* in the etiology of AR Wolfram-like syndrome. The identified variant was submitted to ClinVar (Accession Number: SCV001468320) as a VUS.

## Supplementary information

Supplementary Acknowledgements and Figures

Supplementary Table 1

Supplementary Table 2

Supplementary Table 3
