# Correction: Wolfram-like syndrome with bicuspid aortic valve due to a homozygous missense variant in *CDK13*

**DOI:** 10.1038/s10038-021-00949-3

**Published:** 2021-06-16

**Authors:** Anushree Acharya, Syed Irfan Raza, Muhammad Zeeshan Anwar, Thashi Bharadwaj, Khurram Liaqat, Muhammad Akram Shahzad Khokhar, Jenna L. Everard, Abdul Nasir, University of Washington Center for Mendelian Genomics, Deborah A. Nickerson, Michael J. Bamshad, Muhammad Ansar, Isabelle Schrauwen, Wasim Ahmad, Suzanne M. Leal

**Affiliations:** 1 Center for Statistical Genetics, Gertrude H. Sergievsky Center, and the Department of Neurology, Columbia University Medical Center, New York, NY USA; 2 Department of Biochemistry, Faculty of Biological Sciences, Quaid-i-Azam University, Islamabad, Pakistan; 3 Department of Biochemistry, HBS Medical and Dental College, Islamabad, Pakistan; 4 Department of Biochemistry, CMH Kharian Medical College, Punjab, Pakistan; 5 Major Shabbir Sharif Shaheed Hospital, THQ Level, Kunjah, Gujrat, Punjab Pakistan; 6 Synthetic Protein Engineering Lab (SPEL), Department of Molecular Science and Technology, Ajou University, Suwon, South Korea; 7 Department of Genome Sciences, University of Washington, Seattle, WA USA; 8 Department of Pediatrics, University of Washington, Seattle, WA USA; 9 Taub Institute for Alzheimer’s Disease and The Aging Brain, Columbia University Medical Center, New York, NY USA

**Keywords:** Consanguinity, Genetic linkage study

Correction to: *Journal of Human Genetics*

10.1038/s10038-021-00922-0

The article “Wolfram-like syndrome with bicuspid aortic valve due to a homozygous missense variant in CDK13”, written by Anushree Acharya et al., was originally published electronically on the publisher’s internet portal on 21 April 2021 without open access. With the author’ decision to opt for Open Choice, the copyright of the article changed on 4 June 2021 to ^©^Author(s) 2021 and the article is forthwith distributed under a Creative Commons Attribution 4.0 International License, which permits use, sharing, adaptation, distribution, and reproduction in any medium or format, as long as you give appropriate credit to the original author(s) and the source, provide a link to the Creative Commons licence, and indicate if changes were made. The images or other third party material in this article are included in the article’s Creative Commons licence, unless indicated otherwise in a credit line to the material. If material is not included in the article’s Creative Commons licence and your intended use is not permitted by statutory regulation or exceeds the permitted use, you will need to obtain permission directly from the copyright holder. To view a copy of this licence, visit http://creativecommons.org/licenses/by/4.0.

The original article has been corrected.

