# Supplementary material for: Wolfram-like syndrome with bicuspid aortic valve due to a homozygous missense variant in CDK13
Source: J Hum Genet. 2021 Apr 21;66(10):1009–18. doi: 10.1038/s10038-021-00922-0 (PMC8472924; doi:10.1038/s10038-021-00922-0)
Supplement: Supplementary file 1 — Supplementary Acknowledgements and Figures [file 10038_2021_922_MOESM1_ESM.pdf]

Supplementary Acknowledgements and Figures

Acknowledgements

We also thank the following who provided sequencing at the University of Washington Center for Mendelian Genomics (UW-CMG): Michael J. Bamshad[1,2], Suzanne M. Leal[3], Deborah A. Nickerson[1], Peter Anderson[1], Tamara J. Bacus[1], Elizabeth E. Blue[1], Kati J. Buckingham[1], Jessica X. Chong[1], Diana Cornejo Sánchez[3], Colleen P. Davis[1], Christian D. Frazar[1], Danielle Giroux[1], William W. Gordon[1], Martha Horike-Pyne[1], Jameson R. Hurless[1], Gail P. Jarvik[1], Eric Johanson[1], J. Thomas Kolar[1], Melissa P. MacMillan[1], Colby T. Marvin[1], Sean McGee[1], Daniel J. McGoldrick[1], Betselote Mekonnen[1], Patrick M. Nielsen[1], Karynne Patterson[1], Benjamin Pelle[1], Aparna Radhakrishnan[1], Matthew A. Richardson[1], Gwendolin T. Roote[1], Erica L. Ryke[1], Isabelle Schrauwen[3], Kathryn M. Shively[1], Joshua D. Smith[1], Monica Tackett[1], Machiko S. Threlkeld[1], Gao Wang[3], Jeffrey M. Weiss[1], Marsha M. Wheeler[1], Qian Yi[1], Jordan E. Zeiger[1], and Xiaohong Zhang[1]

Affiliations: [1]University of Washington; [2]Seattle Children's Hospital; and [3]Columbia University. UW-CMG was funded by the National Human Genome Research Institute and the National Heart, Lung and Blood Institute grant HG006493 to Michael J. Bamshad[1,2], Suzanne M. Leal[3], Deborah A. Nickerson[1]**.**

Supplementary Figure 1

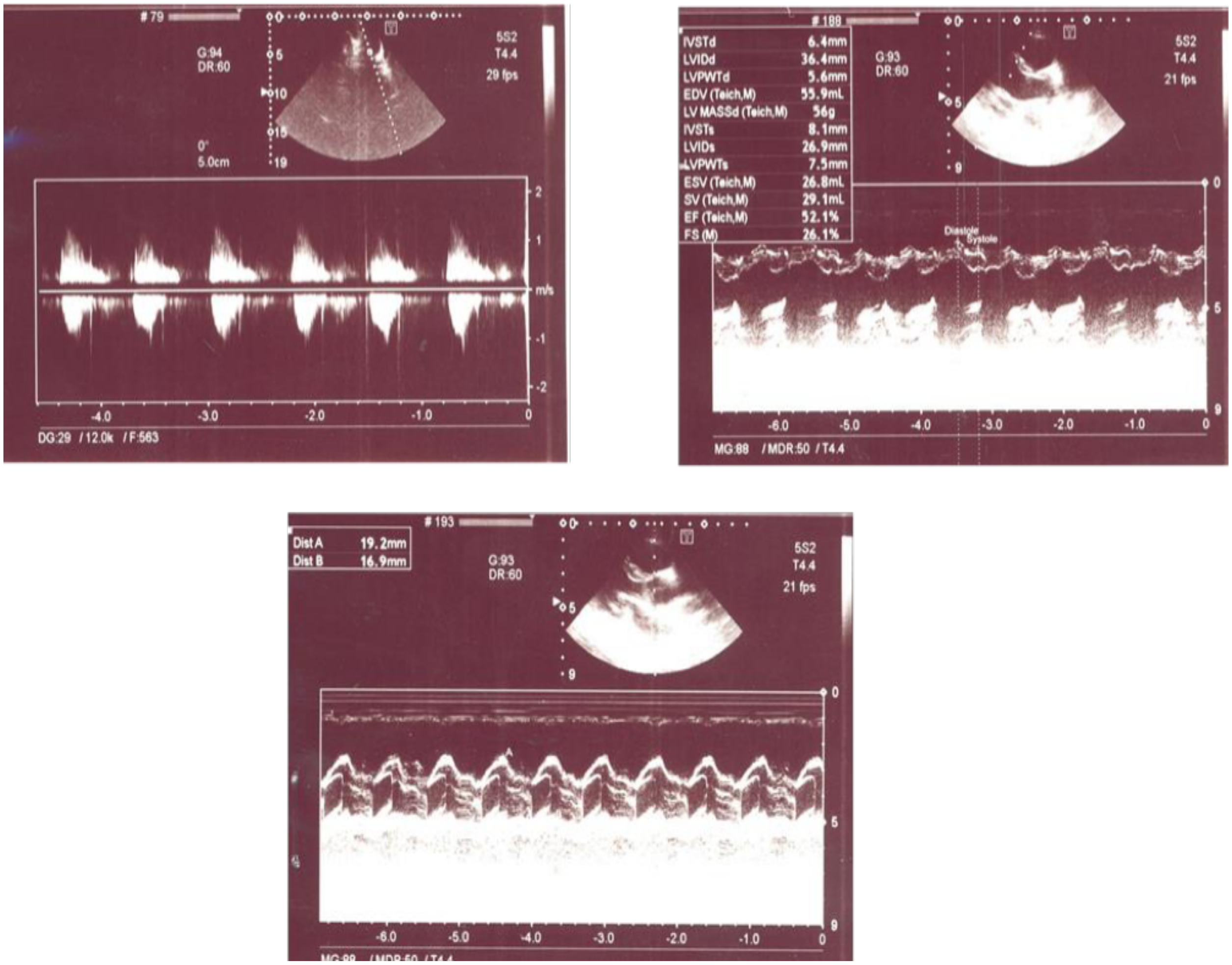

Figure 1A: Echocardiogram of affected child V:3 with bicuspid aortic valve.

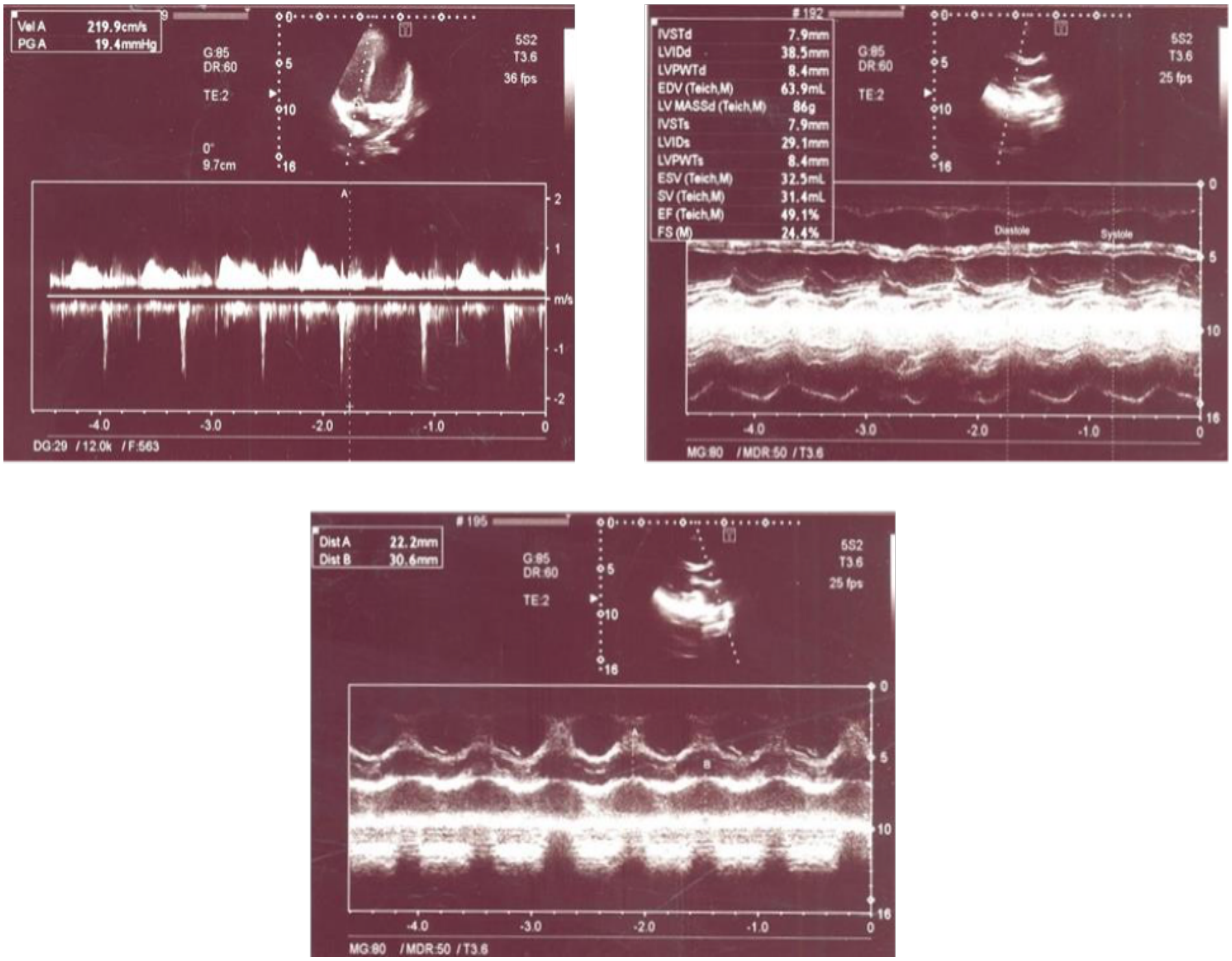

Figure 1B: Echocardiogram of affected child V:4 with bicuspid aortic valve.

Supplementary Figure 2

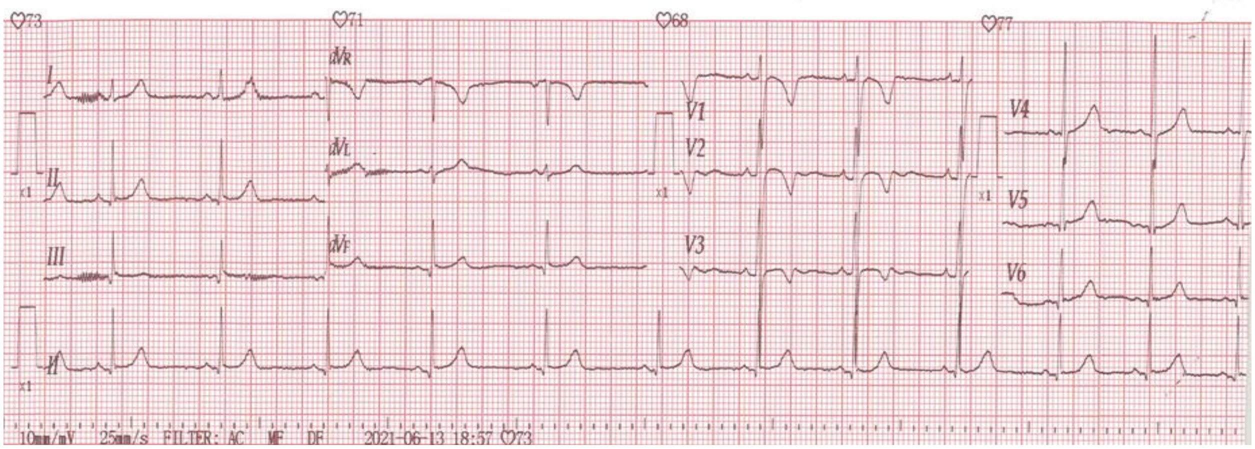

Figure 2A: Normal electrocardiogram of affected child V:3

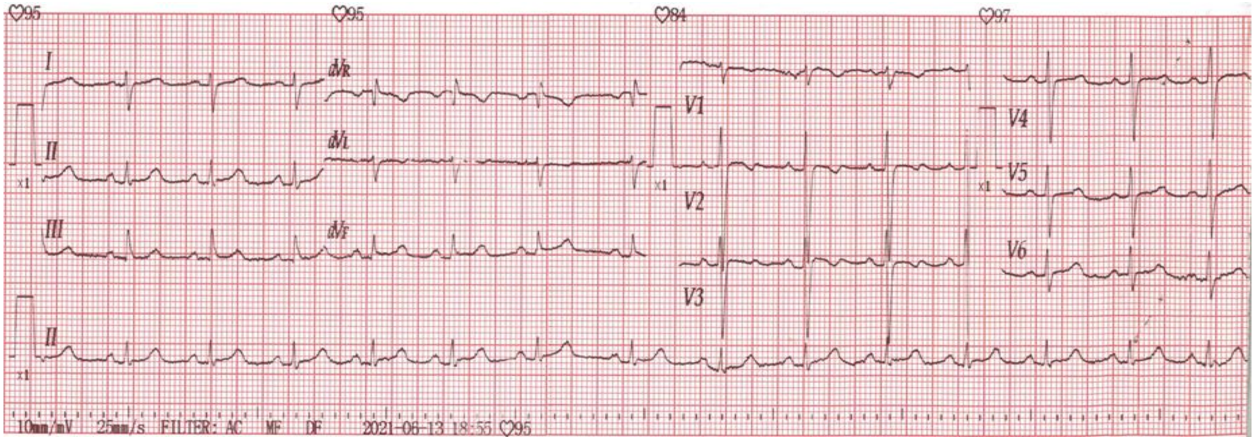

Figure 2B: Normal electrocardiogram of affected child V:4.

Supplementary Figure 3

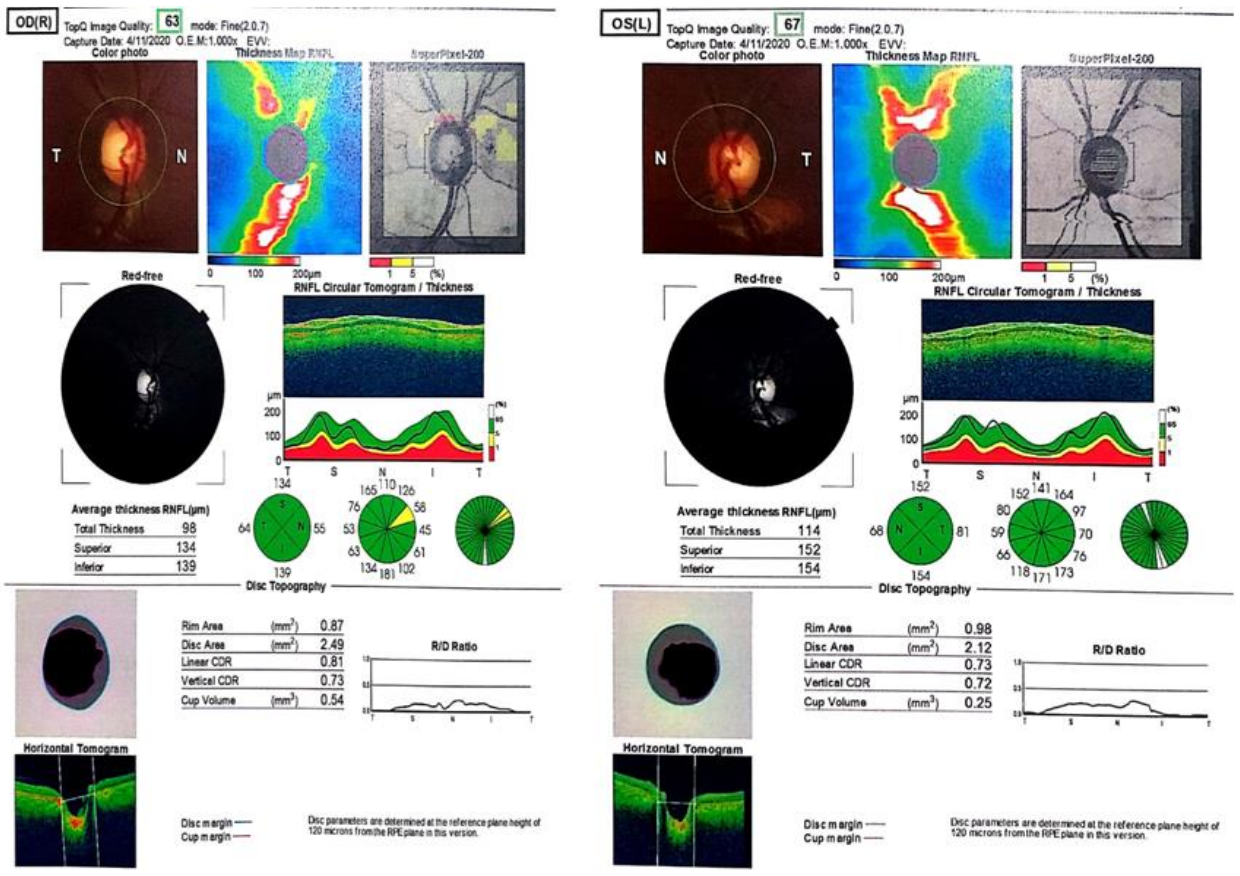

Figure 3: 3D optic disc topography. For the right and left optic nerve heads of affected member V:4 that display normal retinal nerve fiber layer (RNFL) thickness, and optic disc measurements.

Supplementary Figure 4

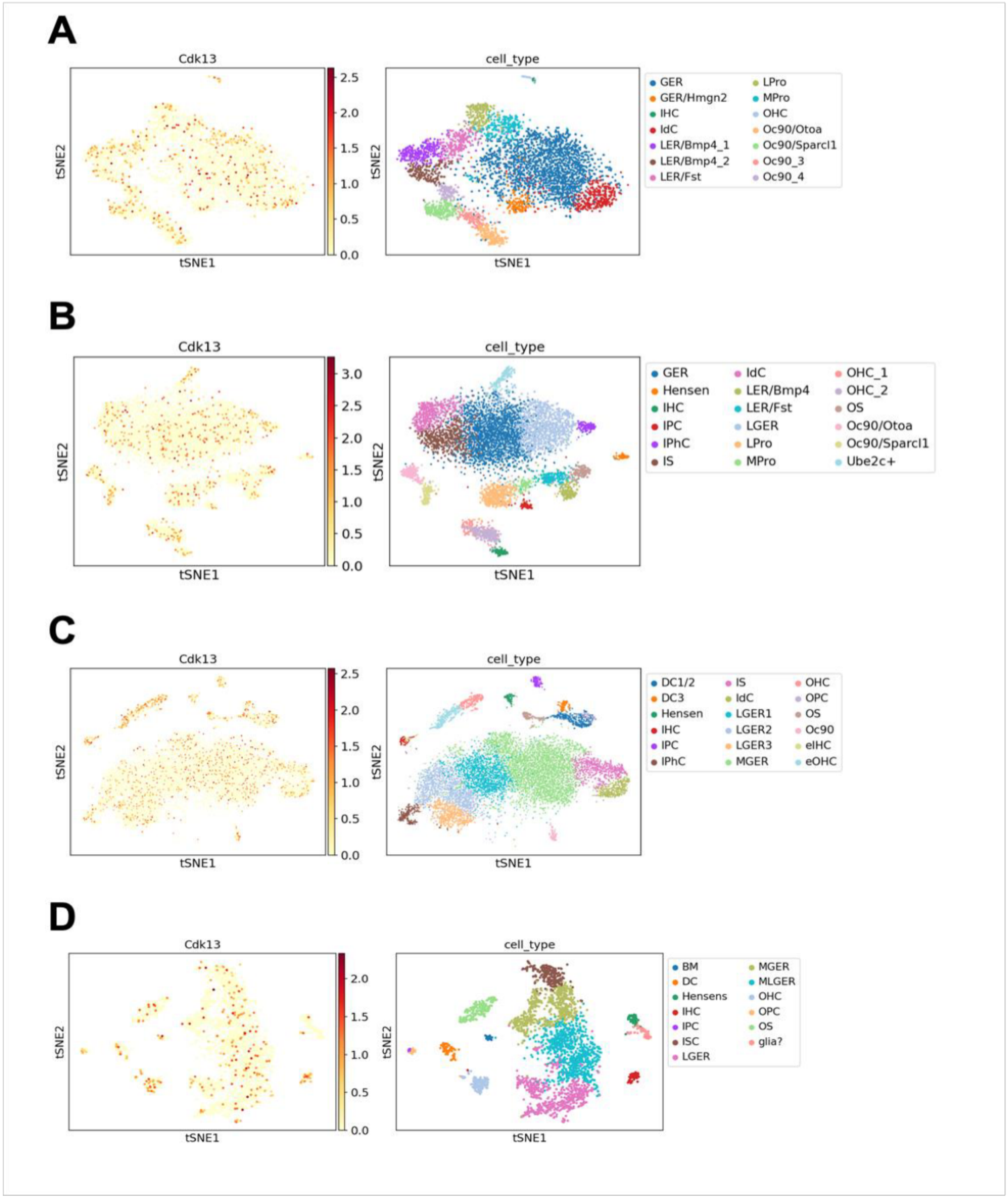

Figure 4: Single cell expression of *Cdk13* during mouse inner ear development*. *Cdk13* expression is shown based on a single-cell RNA sequencing dataset of the cochlear floor

epithelia of CD-1 mice. *Cdk13* expression is shown (left; red = high expression, yellow = low expression) in addition to clustering of cellular types (right; colors indicate the cell type). *Cdk13* expression during mouse development stage: **A.** E14; **B.** E16; **C.** P1; and **D.** P7.

Cdk13 shows a widespread expression in the mouse inner ear during development. Data were obtained from the gEAR database and plots were generated with its analysis suite. The scale bar represents gene expression based on log transformed and normalized expression data.

*Tissue codes: *Developing Supporting Cells*: GER: Greater Epithelial Range; GER/Hmgn2: Greater Epithelial Range expressing Hmgn2; IdC: Interdental Cells; LER/Bmp4_1: Lesser Epithelial Ridge Cells Expressing Bmp4; LER/Bmp4_2: Lesser Epithelial Ridge Cells Expressing Bmp4; LER/Fst: Lesser Epithelial Ridge Cells Expressing Fst; Oc90: Cells expressing Oc90; Oc90/Otoa: Cells expressing Oc90 and Otoa; Oc90/Sparcl1: Cells expressing Oc90 and Sparcl1; Oc90_3: Cells expressing Oc90; Oc90_4: Cells expressing Oc90; OS: Outer Sulcus Cells; IS: Inner Sulcus Cells; IPhC: Inner Phalangeal Cells; IPC: Inner Pillar Cells; OPC: Outer Pillar Cells; Hensen: Hensen Cells; DC: Dieter Cells; DC1/2: Dieter Cells from rows 1 and 2; DC3: Dieter Cells from row 3; BM: Basilar Membrane Cells; Glia: Glial Cells; *Developing Prosensory Cells*: LPro: Lateral Prosensory Cells; MPro: Medial Prosensory Cells. *Developing Greater Epithelial Ridge Cells*: LGER1: Lateral Great Epithelial Ridge Cells, group 1; LGER2: Lateral Great Epithelial Ridge Cells, group 2; LGER3: Lateral Great Epithelial Ridge Cells, group 3; MGER: Medial Greater Epithelial Ridge Cells; MLGER: Medial Lateral Greater Epithelial Ridge Cells; *Developing Sensory Cells*: IHC: Inner Hair Cells; OHC: Outer Hair Cells; OHC_1: More mature developing outer hair cells; OHC_2: Less mature developing outer hair cells. eIHC: Less mature developing inner hair cells; eOHC: Less mature developing outer hair cells; *Uncategorized*: Ube2c+: Unannotated cells expressing Ube2c+.

Supplementary Figure 5

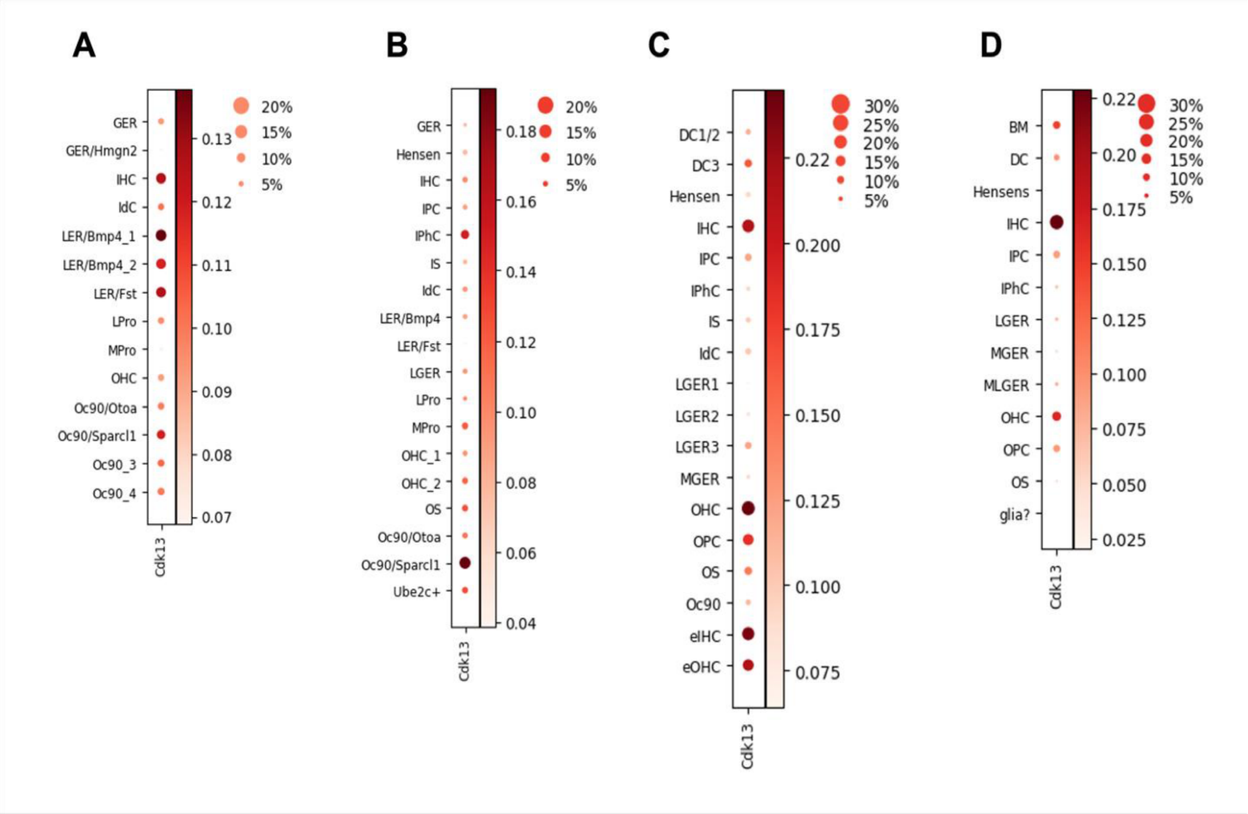

Figure 5: *Cdk13* expression during mouse inner ear development*. Log-transformed and normalized expression levels are indicated for each cell type/cluster from a single cell dataset of the cochlear floor sensory epithelia of developing mice. The dot size indicates the percentage of cells expressing *Cdk13* and the color indicates the intensity of expression (red = highest expression, white = lowest expression). *Cdk13* expression during mouse development stage: **A.** E14; **B.** E16; **C.** P1; and **D.** P7.

These plots illustrate that both the expression intensity of *Cdk13* and the percentage of cells expressing *Cdk13* generally increase as development progresses. There is an especially prominent upregulation of *Cdk13* in IHC from E16 through P7. Upregulation also occurs in the

OHC, yet it ends at P1 and is followed by downregulation through P7. Data was obtained from the gEAR database and plots were generated with its analysis suite.

*Tissue codes: *Developing Supporting Cells*: GER: Greater Epithelial Range; GER/Hmgn2: Greater Epithelial Range expressing Hmgn2; IdC: Interdental Cells; LER/Bmp4_1: Lesser Epithelial Ridge Cells Expressing Bmp4; LER/Bmp4_2: Lesser Epithelial Ridge Cells Expressing Bmp4; LER/Fst: Lesser Epithelial Ridge Cells Expressing Fst; Oc90: Cells expressing Oc90; Oc90/Otoa: Cells expressing Oc90 and Otoa; Oc90/Sparcl1: Cells expressing Oc90 and Sparcl1; Oc90_3: Cells expressing Oc90; Oc90_4: Cells expressing Oc90; OS: Outer Sulcus Cells; IS: Inner Sulcus Cells; IPhC: Inner Phalangeal Cells; IPC: Inner Pillar Cells; OPC: Outer Pillar Cells; Hensen: Hensen Cells; DC: Dieter Cells; DC1/2: Dieter Cells from rows 1 and 2; DC3: Dieter Cells from row 3; BM: Basilar Membrane Cells; Glia: Glial Cells; *Developing Prosensory Cells*: LPro: Lateral Prosensory Cells; MPro: Medial Prosensory Cells. *Developing Greater Epithelial Ridge Cells*: LGER1: Lateral Great Epithelial Ridge Cells, group 1; LGER2: Lateral Great Epithelial Ridge Cells, group 2; LGER3: Lateral Great Epithelial Ridge Cells, group 3; MGER: Medial Greater Epithelial Ridge Cells; MLGER: Medial Lateral Greater Epithelial Ridge Cells; *Developing Sensory Cells*: IHC: Inner Hair Cells; OHC: Outer Hair Cells; OHC_1: More mature developing outer hair cells; OHC_2: Less mature developing outer hair cells. eIHC: Less mature developing inner hair cells; eOHC: Less mature developing outer hair cells; *Uncategorized*: Ube2c+: Unannotated cells expressing Ube2c+.

Supplementary Figure 6

A

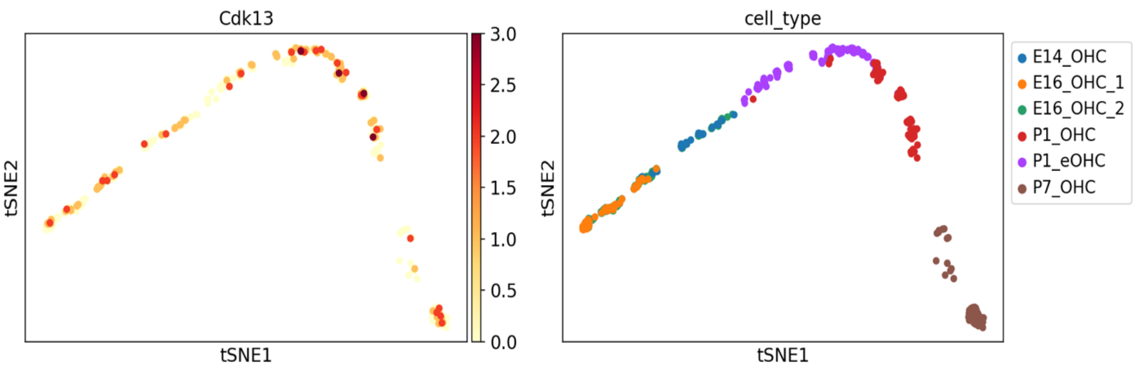

B

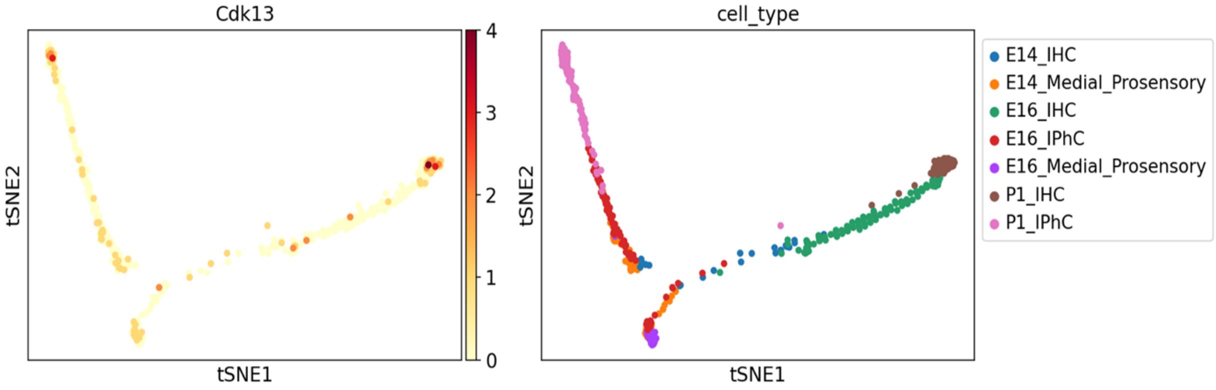

Figure 6: *Cdk13* trajectories in outer hair Cells (OHC) and inner hair cells (IHC)*. *Cdk13* expression is shown based on a single cell dataset of the cochlear floor sensory epithelia of the developing mice, and presented for OHC and IHC. The scale bar represents log-transformed and normalized gene expression level (yellow = low; red = high). **A.** *Cdk13* expression values compared with OHC trajectories during E14-P7. High expression occurs throughout, with the greatest expression in the mature OHC and the least in eOHC during P1. **B.** *Cdk13* expression values compared with IHC, IPhC, and medial prosensory trajectories for E14-P1. Expression of *Cdk13* is greatest in P1 in both IHC and IPhC, exhibiting low expression levels everywhere else.

Data were obtained from the gEAR (gene Expression Analysis Resource) database and plots were generated with its analysis suite.

*Tissue codes: E14_IHC: Early developing inner hair cells E14; E14_Medial_Prosensory: Developing medial prosensory cells E14; E16_IHC: Developing inner hair cells E16; E16_IPhC: Developing inner phalangeal cells E16; E16_Medial_Prosensory: Developing medial prosensory cells E16; P1_IHC: Developing inner hair cells P1; P1_IPhC: Developing inner phalangeal cells P1; E14_OHC: Early developing outer hair cells E14; E16_OHC_1: More mature developing outer hair cells E16; E16_OHC_2: Less mature developing outer hair cells E16; P1_OHC: More mature developing outer hair cells P1: P1_eOHC: Less mature developing outer hair cells P1; P7_OHC: Developing outer hair cells P7.
