# Supplementary material for: Wolfram-like syndrome with bicuspid aortic valve due to a homozygous missense variant in CDK13
Source: J Hum Genet. 2021 Apr 21;66(10):1009–18. doi: 10.1038/s10038-021-00922-0 (PMC8472924; doi:10.1038/s10038-021-00922-0)
Supplement: Supplementary file 2 — Supplementary Table 1 [file 10038_2021_922_MOESM2_ESM.pdf]

Test	Unit	V:3	V:4	Reference Range
Total WBC	/µL	7620	10920	4000-10500
Total RBC	m/µL	5.47	4.74	3.8-5.8
Hemoglobin	g/dL	14.2	12.8	12.0-15.0
HCT	%	40.6	37.2	35-45
MCV	fL	74.2	78.5	78-95
MCH	pg	26	27	26-32
MCHC	g/dL	35	34.4	32-36
Platelet Count	/µL	403000	479000	150000-400000
Neutrophils	%	32	49	50-60
Lymophocytes	%	54	34	35-40
Monocytes	%	11	9	1-4
Eosinophils	%	3	8	1-2
Basophil	%	0	0	0-0.75
RDW	%	12.7	13.5	11.5-14.0
MPV	fL	9.8	8.6	6.8-10.2
HbA1C	%	10.6	10.7	<5.7% - Normal; 5.7-6.4%- Pre-diabetic; >= 6.5% Diabetes mellitus
D-Dimer	mg/L	0.29	0.15	upto 0.50mg/L
Liver Function Tests-				
SGOT (AST)	U/L	22	11	upto 32
SGPT (ALT)	U/L	18	13	upto 33
Alkaline Phosphatase	U/L	242	110	Children: 11-15y upto 468 U/L
Total Bilirubin	mg/dL	0.37	<0.15	upto 1.2mg/dL
Direct Bilirubin	mg/dL	0.12	<0.08	upto 0.30
Gamma G.T	U/L	15	13	upto 40
Total Protein	g/dL	NA	7.4	6-8.5
Albumin	g/dL	NA	3.7	3.5-5
Globulins	g/dL	NA	3.7	1.8-3.2
Albumin:Globulins		NA	1	1.2-2.2
tTG-IgA	U/L	NA	28	upto 15
Renal Function -				
Urea	mg/dL	NA	22	10-50
Creatinine	mg/dL	NA	0.5	0.5-1.5

Serum Electrolytes -

Sodium	mmol/L	NA	140	136-145
Potassium	mmol/L	NA	3.7	3.5-5.4
Chloride	mmol/L	NA	107	95-108
Bicarbonate	mmol/L	NA	21	21-32

HCT = Hematocrit
MCV= Mean corpuscular volume
MCH = Mean corpuscular hemoglobin
MCHC = Mean corpuscular hemoglobin concentration
RDW = Red cell distrubution width
MPV = Mean platelet Volume
HbA1c = Hemoglobin A1c
SGOT (AST) = Serum glutamic-oxaloacetic transaminase (Aspartate transaminase)
SGPT (ALT) = Serum glutamic pyruvic transaminase (Alanine transaminase)
tTG-IgA = Tissue transglutaminase immunoglobulin A
NA = Not available
