# Supplementary material for: Wolfram-like syndrome with bicuspid aortic valve due to a homozygous missense variant in CDK13
Source: J Hum Genet. 2021 Apr 21;66(10):1009–18. doi: 10.1038/s10038-021-00922-0 (PMC8472924; doi:10.1038/s10038-021-00922-0)
Supplement: Supplementary file 3 — Supplementary Table 2 [file 10038_2021_922_MOESM3_ESM.pdf]

Homozygous regions in genotype data of all 6 members

**Chr**	**hg19_start**	**hg19_end**	**Score**
3	9,091,946	22,694,778	750
7	37,213,483	45,963,114	750
7	106,157,161	130,629,493	750
10	54,832,274	82,452,030	750
11	69,052,682	70,374,786	661
